# Supplementary material for: Temporal genomics in Hawaiian crickets reveals compensatory intragenomic coadaptation during adaptive evolution
Source: Nat Commun. 2024 Jun 12;15:5001. doi: 10.1038/s41467-024-49344-4 (PMC11169259; doi:10.1038/s41467-024-49344-4)
Supplement: Supplementary file 1 — Supplementary information [file 41467_2024_49344_MOESM1_ESM.pdf]

Supplementary information for:

Temporal genomics in Hawaiian crickets reveals compensatory intragenomic coadaptation during adaptive evolution

Xiao Zhang[1,2,*], Mark Blaxter[3], Jo Wood[3], Alan Tracey[3], Shane McCarthy[3], Peter Thorpe[4,5], Jack G. Rayner[2], Shangzhe Zhang[2], Kirstin L. Sikkink[6], Susan L. Balenger[7], Nathan W. Bailey[2,*]

[1] Tianjin Key Laboratory of Conservation and Utilization of Animal Diversity, College of Life Sciences, Tianjin Normal University, Tianjin, 300387, China.

[2]Centre for Biological Diversity, School of Biology, University of St Andrews, St Andrews, Fife, KY16 9TH, UK.

[3]Tree of Life, Wellcome Sanger Institute, Cambridge CB10 1SA, UK.

[4]School of Medicine, University of St Andrews, St Andrews, Fife, KY16 9TF, UK.

[5]Present address: School of Life Sciences, University of Dundee, Dundee, DD1 4HN, UK.

[6]Arima Genomics, Carlsbad, CA, 92011, USA.

[7]College of Biological Sciences, University of Minnesota, Saint Paul, MN, 55108, USA.

[*]**e-mail:** xz42@st-andrews.ac.uk; nwb3@st-andrews.ac.uk

Table of contents

Supplementary Figs. 1-10

Supplementary Tables. 1-12

Supplementary Note

Supplementary References

*Supplementary Data 1-8 are in separate Excel files

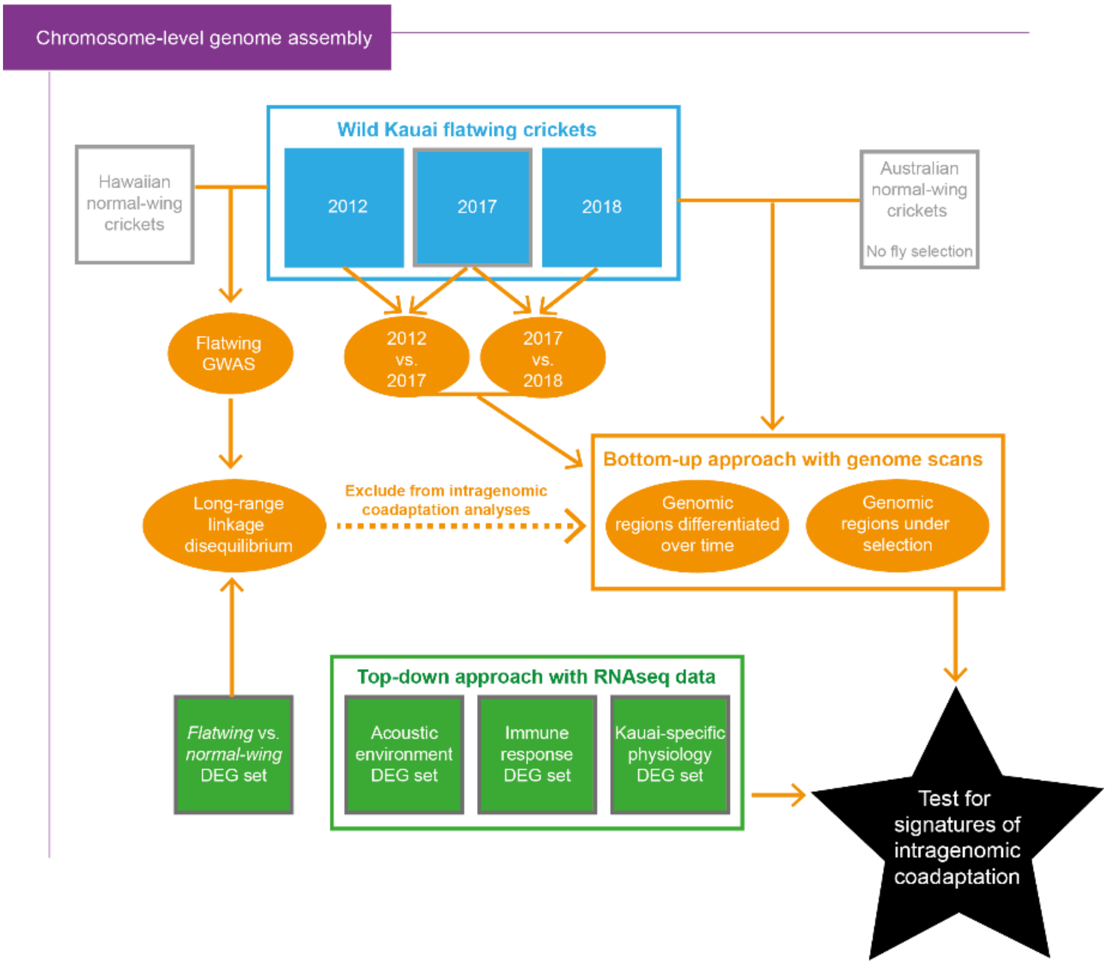

Supplementary Figure 1. Schematic workflow for this study summarizing the core goal, resource development, data sources, methodological procedures, and interrelations. *Goal*: The overall scientific goal of the study is indicated by the black star which describes a synthetic analysis of bottom-up and top-down approaches. *Resources*: Purple rectangle indicates annotated reference genome resource developed in this study. *Data*: Squares indicate sequence data utilized in this study. Those shaded in blue denote whole-genome resequencing (WGRS) data from field sampling in Kauai, and icons outlined in grey represent previously published data. Green shading represents differentially expressed genes obtained in this study from previously-published reads (see Fig. 5 and Supplementary Note for details). *Procedures*: Orange ovals indicate important intermediate steps and contrasts. Arrows show connections between data, experimental procedures, and conclusions.

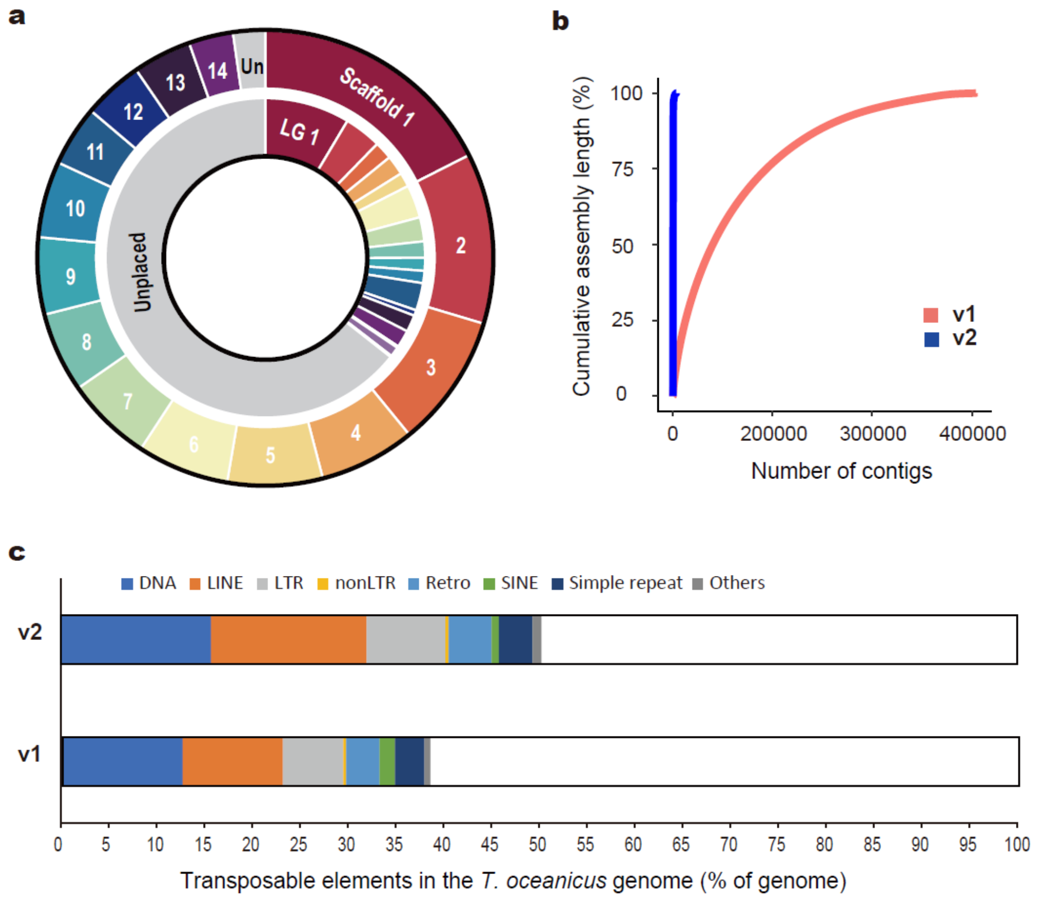

Supplementary Figure 2. Comparison of basic features of two *T. oceanicus* genome assemblies. a. Circos plot providing an overview of the chromosome-level scaffolds (v2) assembled using the ONT sequencing and Hi-C technologies (outer track) and previously reported linkage groups upon which scaffolds of the v1 assembly were anchored. Non-chromosome-level scaffolds of the v2 assembly and unplaced scaffolds of the v1 assembly are shown in gray. **b.** Cumulative contig plots of the original *T. oceanicus* genome assembly (v1) and the new assembly (v2). The high contig-level contiguity of this new assembly is visualized by the near vertical line representing very long contigs. **c.** Proportions of seven major categories of transposable elements detected in the new *T. oceanicus* genome assembly (v2) contrasted with the original assembly (v1).

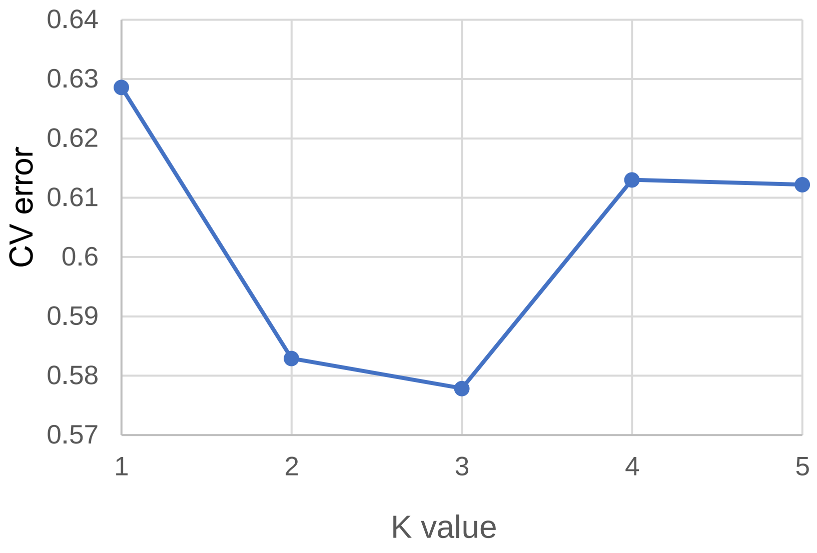

Supplementary Figure 3. Cross-validation plot suggesting the best K value.
Source data are provided as a Source Data file.

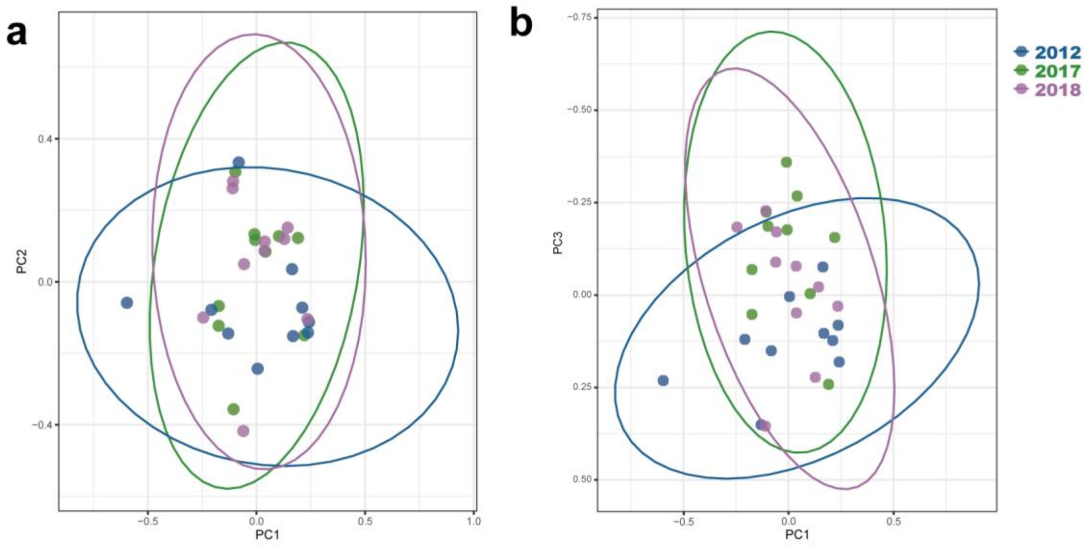

Supplementary Figure 4. Two-dimensional principal component (PC) plots showing genomic distance of wild Kauai samples across years. a. PC 1 (x-axis) vs. PC 2 (y-axis) **b.** PC 1 (x-axis) vs. PC 3 (y-axis). Blue = Kauai individuals sampled in 2012, green = Kauai individuals sampled in 2017, purple = Kauai individuals sampled in 2018. Source data are provided as a Source Data file.

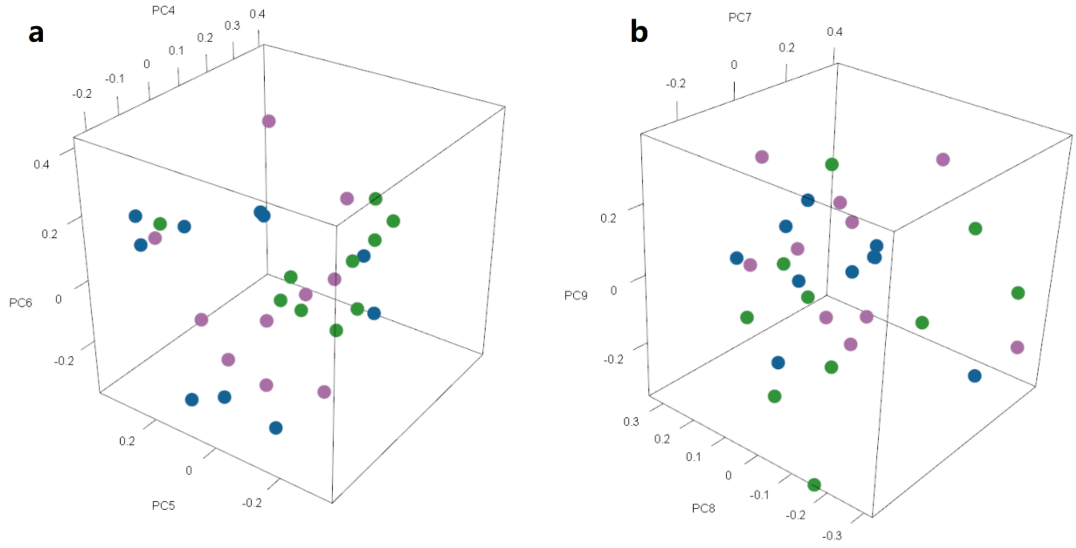

Supplementary Figure 5. Principal component (PC) plots showing genomic distance of Kauai samples. **a.** Principal components 4 – 6 **b.** Principal components 7-9. Genomic variance explained for PC 4: 1.59%, PC 5: 1.53%, PC 6: 1.40%, PC7: 1.28%, PC 8: 1.16%; PC 9: 1.03%. Blue = Kauai individuals sampled in 2012, green = Kauai individuals sampled in 2017, purple = Kauai individuals sampled in 2018. Source data are provided as a Source Data file.

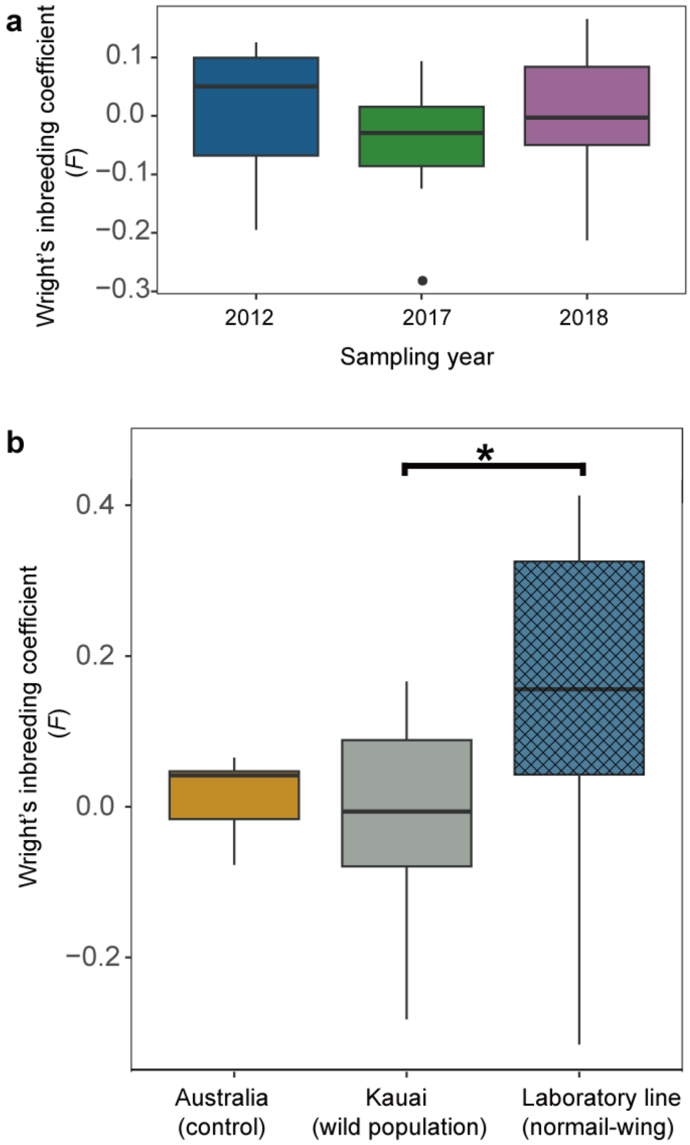

Supplementary Figure 6. Comparisons of Wright's inbreeding coefficient for three time points in wild-caught Kauai flatwing crickets, plus two control groups. a. The inbreeding coefficient for each sampling year remains stable within the Kauai population. **b.** Data from the wild outbred Australian population and the inbred normal-wing laboratory stock originally derived from Kauai were used for comparison. The inbreeding laboratory line showed significantly higher *F* values than wild flatwing individuals. Horizontal lines indicate medians, boxes interquartile ranges, and whiskers the data range. Asterisk indicates significance at $P < 0.05$, n = 10 individuals for each sampling year, n = 7 individuals for Australia, n = 30 individuals for Kauai, n = 10 individuals for laboratory line, see Main Text for details. Exact *P* values and additional statistical details are provided in Supplementary Table 7. Source data are provided as a Source Data file.

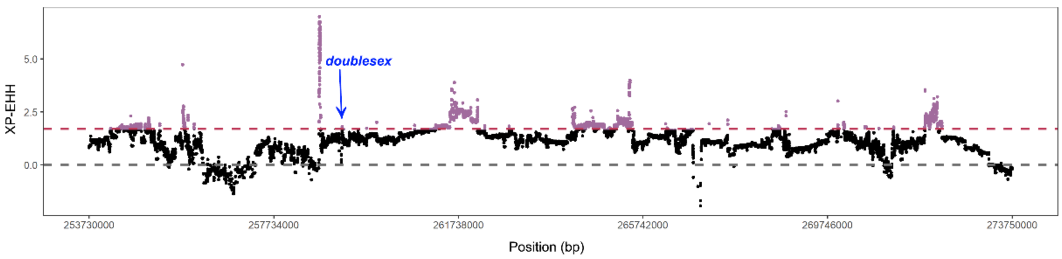

Supplementary Figure 7. Distribution of standardized cross-population extended haplotype homozygosity (XP-EHH) scores in the highly-linked, flatwing-associated region of the X chromosome containing *doublesex*. The black horizontal dashed line indicates the mean background value for XP-EHH scores, while the red horizontal dashed line indicates the top 5% threshold of XP-EHH scores. The scores passing the top 5% are plotted in purple.

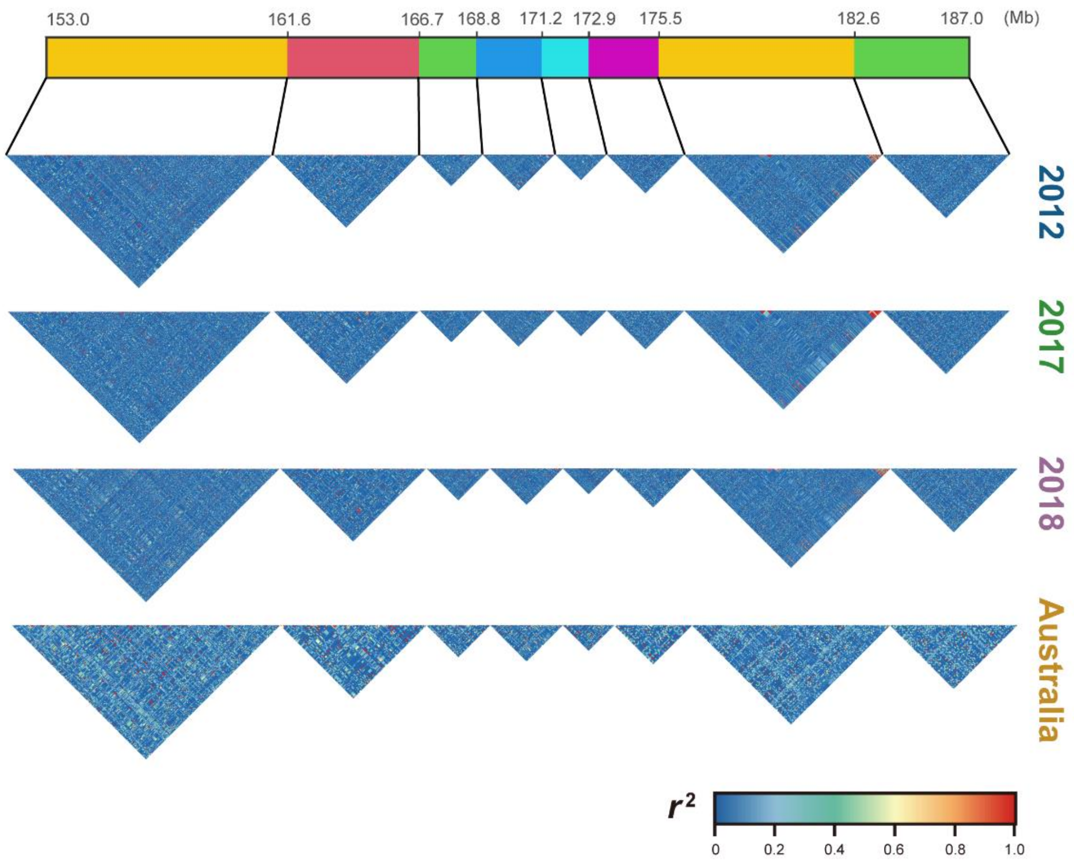

Supplementary Figure 8. Linkage disequilibrium along a randomly chosen region of the X chromosome of similar length to the linkage block containing *flatwing* reported in the Main Text. The same three time points of the evolutionary time-series are compared. Each contiguous block in the LD pattern corresponds to a contig to avoid any visual bias introduced by gaps in the genome assembly.

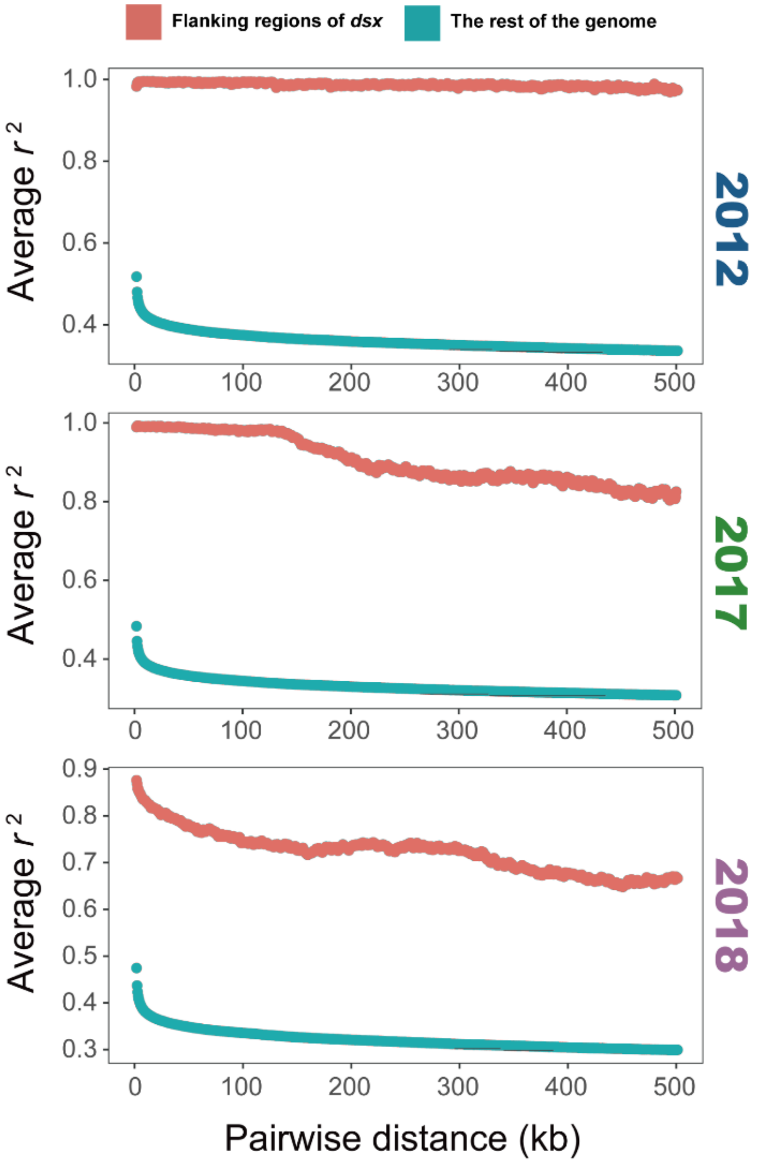

Supplementary Figure 9. Decay patterns of linkage disequilibrium flanking the candidate *flatwing* region containing the *dsx* locus (red) compared to genomic background (blue) illustrated by mean pairwise r^2 values for each sampled timepoint of the wild Kauai population.

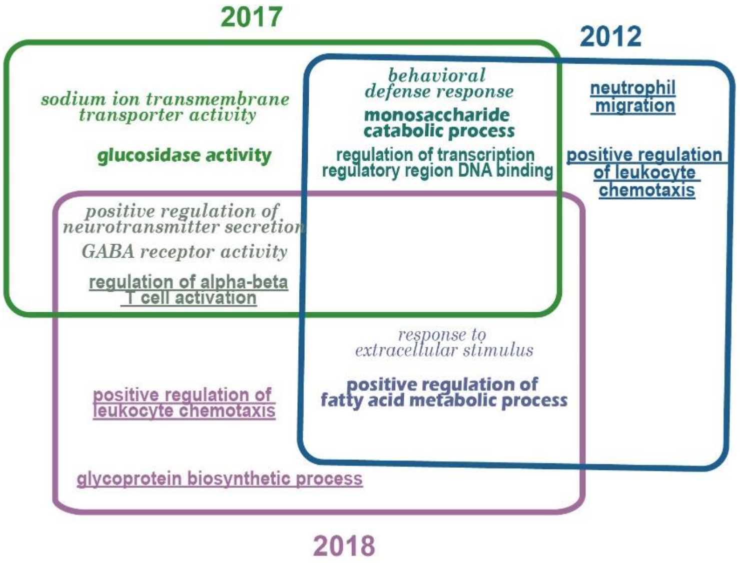

*GO terms are classified into four groups(*behavior-related*, immune response-related, **locomotion-related**, other important traits)

Supplementary Figure 10. Venn-like diagram presenting the canonical Gene Ontology (GO) functional annotations that are significantly enriched (FDR-adjusted *P* < 0.05) in the selective genomic regions. The DEG sets, GO, and KEGG terms are categorized into four groups: behavior-related, immune response-related, locomotion-related, and other important traits. These categories are indicated by different typefaces, as shown in the brackets.

Supplementary Table 1. Summary of the chromosome-level assembly for *T. oceanicus*.

Genome metrics	Value
Genome size (bp)	2028946150
Scaffold number	1830
Number of gaps	1521
Length of the largest scaffold (bp)	355482399
Scaffold N50 (bp)	137364004
Contig N50 (bp)	5709036

Supplementary Table 2. Comparison of basic genome metrics among seven cricket genome assemblies.

		Size (Gb)	Contig N50 (kb)	Maximum contig length (Mb)	Scaffold N50 (kb)	Maximum scaffold length (Mb)	Chromosome-level assembly	BUSCO (%)	Ref.
T. oceanicus	v 2	2.0	5709.0	23.1	143,560	372.92	98%	98	This study
	v 1	2.0	13.3	0.2	63	2.64	×	94	1
T. occipitalis		1.9	197.5	2.2	214	2.30	×	98	2
Laupala kohalensis		1.6	42.9	0.5	583	4.54	×	99	3
Gryllus bimaculatus		1.7	31.7	0.4	6,287	29.75	×	99	4
Acheta domesticus		2.2	321.1	-	221,839	304.00	84%	95	5
Apteronemobius asahinai		1.7	26.4	0.9	27.0	0.93	×	92	6

Supplementary Table 3. BUSCO analysis of the chromosome-level genome assembly.

Type	Number	Percentage
Complete BUSCOs (C)	988	97.6%
Complete and single-copy BUSCOs (S)	963	95.1%
Complete and duplicated BUSCOs (D)	25	2.5%
Fragmented BUSCOs (F)	5	0.5%
Missing BUSCOs (M)	20	1.9%
Total BUSCO groups searched	1013	100.0%

Supplementary Table 4. Comparison of gene features among five cricket genome assemblies.

	T. oceanicus (v2)	*T. oceanicus* (v1)	*T. occipitalis*	*Gryllus bimaculatus*	*Laupala kohalensis*
Number of gene	21,211	19,157	20,768	17,871	12,767
Number of cds	29,835	28,246	20,768	28,529	13,078
Number of exon	203,721	173,806	114,824	233,361	89,797
Number of single exon gene	2,588	1,266	5,478	1,097	1,088
mean exons per mrna	7	6	6	8	7
Total gene length	565,164,176	234,238,894	307,934,823	531,333,526	168,895,025
Total cds length	37,653,663	33,447,196	28,189,698	38,757,552	17,662,773
Total exon length	64,810,392	68,583,100	28,189,698	74,821,767	18,286,906
mean gene length	26,644	12,227	14,827	29,731	13,229
mean exon length	318	394	245	320	203

Supplementary Table 5. Analysis of variance (ANOVA) results examine the difference of nucleotide diversity across years.

	Df	Sum Sq	Mean Sq	F value	*P*
Year	2	0.00001	5.58E-06	0.455	0.634
Residuals	4866	0.05964	1.23E-05		

Supplementary Table 6. Analysis of variance (ANOVA) results examine the difference of Wright's inbreeding coefficient (*F*) between Australian and Kauai individuals across years.

	Df	Sum Sq	Mean Sq	F value	*P*
Group	3	0.0218	0.007257	0.642	0.593
Residuals	33	0.3729	0.011299		

Supplementary Table 7. Fisher's Least Significant Difference test examine the difference of Wright's inbreeding coefficient (*F*) among Australian, Kauai wild-collected and lab inbreeding individuals.

	Df	Sum Sq	Mean Sq	F value	*P*
Group	2	0.1581	0.07903	3.836	0.0291*
Residuals	44	0.9067	0.02061		
	ntr	alpha			
Fisher-LSD	3	0.05			

Supplementary Table 8. Five key genes located in the highly linked region and exhibited heightened expression levels in Kauai flatwing individuals.

Gene ID	Location		Gene name	UniProt ID
TOSG006534	Scaffold_1	264130019 - 264179289	*Sh*	KCNAS
TOSG006538	Scaffold_1	264348611 - 264838552	*NaCP60E*	SCN60
TOSG019074	Scaffold_1	256564040 - 256629216	*Mfsd14a*	MF14A
TOSG019068	Scaffold_1	256922808 - 256926129	*HSD17B12*	DHB12
TOSG019067	Scaffold_1	256976135 - 257021248	*BMI1*	BMI1

Supplementary Table 9. Summary of putative genomic regions under selection in the Kauai population

		**2012**	**2017**	**2018**	**2012 vs 2017**	**2017 vs 2018**
Autosome	Total region size (Mb)	39.63	37.80	38.49	8.44	19.69
	Number of selective-sweep regions	2255	2174	2134	392	773
	Number of candidate genes	779	744	781	171	349
Chr X	Total region size (Mb)	0.50	0.58	0.98	3.62	0.71
	Number of selective-sweep regions	42	49	84	133	46
	Number of candidate genes	6	11	15	19	11
Whole genome	Total region size (Mb)	40.14	38.38	39.48	12.06	20.41
	Number of selective-sweep regions	2297	2223	2218	525	819
	Number of candidate genes	785	755	796	190	360

Supplementary Table 10. Validation of putative genes under selection using phased-genotype-based approaches (iHS and XP-EHH; see Main Text for details).

	2012	2017	2018	2012 vs 2017	2017 vs 2018	Summary
Candidate gene under selection with phased genotypes	561	597	606	289	145	2198
Candidate genes supported by additional selection statistics	430	455	463	176	93	1617
Verification rate (%)	77	76	76	61	64	74

Supplementary Table 11. Summary of enrichment tests for the genes under selection and associated with responses to fly infestation.

Year	Fisher Extract test *P* value	Increased gene ratio (%)
2012	0.000760456	5.45
2016	0.023122214	3.64
2017	0.027562031	3.64

Supplementary Table 12. Summary of genes differentiated between years and associated with the Kauai population's unique physiological response to fly infestation.

	Group	
	2012 vs 2017	2017 vs 2018
Genes associated with the Kauai population's unique physiological response	4	25
The other genes differentiated between years	186	335
Total number	190	360

*X-squared = 4.9022, df = 1, *P* value = 0.02682

Supplementary Note

Details of RNA-seq datasets and analyses

Here we briefly summarize the designs of studies from which RNAseq data were obtained and describe how they were used in this study. Data from 114 *T. oceanicus* RNA-seq libraries were obtained from the European Nucleotide Archive (ENA), where they had been deposited in connection with five previous studies (Supplementary Data 2). These datasets covered four tissues (forewing buds of developing nymphs, embryo thoracic tissue[1], brains i.e. tissue contained within the head capsule, and body tissues), three populations (Kauai and Oahu in Hawaii, and Mangaia in the Cook Islands), both sexes (male and female), and two wing morph genotypes (*normal-wing* and *flatwing*). Data from all 114 RNA-seq libraries were used for gene prediction during genome annotation.

Data from specific contrasts were used to identify candidate differentially-expressed genes (DEGs) that we then used to test for evidence of compensatory intragenomic coadaptation over the course of *flatwing's* spread to fixation and resultant altered social environment of the studied Kauai population. These candidate DEGs represented specific components of such compensatory adaptation, as follows.

Candidate DEGs directly impacted by genomic invasion of flatwing

Libraries derived from dorsal right forewing buds[7] and embryo thoracic tissue[1] were used to reconfirm the important roles of the top *flatwing*-associated candidate gene *doublesex* and highlight candidates in the highly-linked flanking region of *doublesex* whose expression was directly perturbed by the genomic invasion of the *flatwing* variant; the key contrasts were expression profiles of *normal-wing* vs. *flatwing* samples. For this task, we used 12 dorsal right forewing bud libraries, and 12 embryonic thoracic libraries from two independent studies[1,7]. Samples for both studies were collected from similar lab-reared, pure-breeding lines of *T. oceanicus* produced by performing two generations of standard crosses with mixed stock derived from Kauai (as described in [8]). As the phenotypic manifestation of *flatwing* is confined to one sex, F_2 male offspring were used to identify and isolate homozygous *flatwing* and homozygous *normal-wing* lines. This screening process yielded three pure-breeding lines for *flatwing* and *normal-wing* genotypes. As

flatwing is on the X chromosome, all individuals of these biological lines had either homozygous (hemizygous if male) *normal-wing* or *flatwing* genotypes. Hereafter, these two studies containing both flatwing and normal-wing Kauai individuals are referred to as "*wing bud transcriptome*" and "*embryonic thoracic transcriptome*" respectively. *Wing bud transcriptome* used five individuals per pool and 3 samples per group sequenced on an Illumina HiSeq 4000 [7]. We used this *wingbud transcriptome* dataset to confirm the previously reported differential expression patterns of *doublesex* between individuals carrying *flatwing* and *normal-wing* variants [7].

For the 12 embryonic thoracic samples, RNA extraction was also performed on Kauai lines homozygous for either the *flatwing* or *normal-wing* genotypes [8]. As described in Pascoal et al. [1], developing embryos were collected from eggs laid by females that were isolated and allowed to oviposit after mating. Eggs were retrieved from oviposition substrates and promptly preserved in RNAlater at a developmental stage approximately two weeks post-laying. Following removal of the outer egg chorion, the thoracic segment of each nymph was subjected to microdissection. To mitigate potential variance in sex ratios across samples between lines and to ensure an adequate tissue volume for RNA extraction, thoracic tissues from 8 nymphs were pooled for each replicate, resulting in the production of six biological replicates for each genotype (i.e. two samples per line). This dataset was used to highlight the genes involved in previously-reported cascade of downstream regulatory effects associated with *flatwing* [1].

Candidate DEGs related to secondary adaptation to the altered social environment

Next, we used RNA-seq datasets from experimental work that exposed crickets to novel social conditions (silence) which was evolutionarily caused by the original, direct source of selection (lethal flies eavesdropping on male song) to identify candidate genes associated with responses to the transition from a singing to a silent population in Kauai. Put another way, this procedure identified candidate loci experiencing compensatory intragenomic coadaptation during the rapid evolution of silence. We used brain samples of crickets subjected to experimentally manipulated silence vs. normal levels of male song in their environment to detect candidate differentially expressed genes related to the changed acoustic environment.

As above, Kauai pure-breeding *flatwing* and *normal-wing* lines were used [9]. Twenty-four samples were divided into two treatment groups. One group was kept in silence mimicking a population with no normal-wing males. The other group was exposed to two different Kauai male calling song playbacks, mimicking a population with a high density of singing males[9,10]. As described in Pascoal et al. [9], stock crickets were reared in 16 L plastic containers under common

garden conditions. Upon the emergence of sex differences during nymphal development males and females were individually isolated into 118 mL plastic cups to ensure virginity and control pre-adult social interactions. To prevent interference with the experimental treatment, wing scrapers were removed from all crickets to prevent singing after adult eclosion.

These isolated crickets were then randomly allocated to one of four incubators. To manipulate the acoustic environment perceived by the crickets, two incubators remained silent ('no song' treatment), simulating a population lacking normal-wing males, while the other two played back male calling songs reflecting average song parameters of Kauai males ('song' treatment). The latter simulated the ancestral condition of a high-density of singing, normal-wing males. Song playback occurred exclusively during the crickets' nocturnal phase. After one week of adulthood in their respective treatments, cricket head capsule tissues were dissected and preserved in RNALater for subsequent RNA extraction. Samples were sequenced on a HiSeq 2000 platform[9].

Candidate DEGs related to cricket immune responses to parasitoid infestation

We utilized RNA-seq data obtained from a study[11] of 12 parasitoid fly-infested cricket bodies and 6 uninfected controls to identify genes associated with immune responses provoked by *Ormia ochracea* infestation, as well as those reflecting responses unique to the Kauai population as compared with a naïve, unparasitized cricket population. The rationale was to identify candidate DEGs related to ongoing selection that is known not to have changed during the fixation of flatwing males in this population: *O. ochracea* persists in Kauai and continues to infest crickets despite the lack of singing males[12,13].

The study by Sikkink et al. (2020)[11] used one cricket stock colony originating from the same wild Kauai population as above, plus another stock colony from Mangaia in the Cook Islands (−21°55'S −157°55'W). Mangaia crickets exhibit a normal-wing phenotype, and parasitoid flies are not detected on the island. Flies utilized in the study were sourced from the Santa Monica Mountains, Los Angeles County, California, due to logistical constraints in obtaining gravid flies from Hawaii. Mitochondrial haplotype and microsatellite genotype analyses suggest a Western U.S. origin for *O. ochracea* populations across the Hawaiian Islands [14]. Following established protocols[15], sexually mature adult male crickets were artificially infested with *O. ochracea*, with two planidia applied to simulate natural infestation levels. Infested crickets were then isolated in individual containers within an incubator for 24 hours, while control crickets underwent similar handling without larval transfer. Tissue collection occurred either four or seven days post-infestation based on behavioral studies indicating significant changes in courtship and reproductive

behaviors at these time points. Crickets from which larvae naturally emerged between six and seven days post-infestation were excluded. Cricket head and body tissues were segregated and the digestive tract discarded. Tissue samples, excluding the digestive tract, were preserved in RNAlater Solution before RNA extraction. Pronota, wings, and legs were removed from bodies, while larvae were dissected from the abdomen before RNA extraction. RNA was extracted from cricket bodies, with total RNA pooled prior to library preparation to ensure equal representation of samples from two individuals of the same treatment group. Subsequently, 18 pooled samples were multiplexed and sequenced on an Illumina HiSeq 2000 platform.

Supplementary References

1. Pascoal, S. *et al.* Field cricket genome reveals the footprint of recent, abrupt adaptation in the wild. *Evol. Lett.* **4**, 19-33 (2020).
2. Kataoka, K. *et al.* The draft genome dataset of the Asian cricket *Teleogryllus occipitalis* for molecular research toward entomophagy. *Front. Genet.* **11**, 470 (2020).
3. Blankers, T., Oh, K. P., Bombarely, A. & Shaw, K. L. The genomic architecture of a rapid island radiation: recombination rate variation, chromosome structure, and genome assembly of the Hawaiian cricket *Laupala*. *Genetics* **209**, 1329-1344 (2018).
4. Ylla, G. *et al.* Insights into the genomic evolution of insects from cricket genomes. *Commun. Biol.* **4**, 733 (2021).
5. Dossey, A. T. *et al.* Genome and genetic engineering of the house cricket (*Acheta domesticus*): a resource for sustainable agriculture. *Biomolecules* **13** (2023).
6. Satoh, A., Takasu, M., Yano, K. & Terai, Y. De novo assembly and annotation of the mangrove cricket genome. *BMC Res. Notes* **14**, 387 (2021).
7. Zhang, X., Rayner, J. G., Blaxter, M. & Bailey, N. W. Rapid parallel adaptation despite gene flow in silent crickets. *Nat. Commun.* **12**, 50 (2021).
8. Pascoal, S. *et al.* Rapid evolution and gene expression: a rapidly evolving Mendelian trait that silences field crickets has widespread effects on mRNA and protein expression. *J. Evol. Biol.* **29**, 1234-1246 (2016).
9. Pascoal, S. *et al.* Increased socially mediated plasticity in gene expression accompanies rapid adaptive evolution. *Ecol. Lett.* **21**, 546-556 (2018).
10. Pascoal, S. *et al.* Sexual selection and population divergence I: the influence of socially flexible cuticular hydrocarbon expression in male field crickets (*Teleogryllus oceanicus*). *Evolution* **70**, 82-97 (2016).
11. Sikkink, K. L., Bailey, N. W., Zuk, M. & Balenger, S. L. Immunogenetic and tolerance strategies against a novel parasitoid of wild field crickets. *Ecol. Evol.* **10**, 13312-13326 (2020).
12. Zuk, M., Bailey, N. W., Gray, B. & Rotenberry, J. T. Sexual signal loss: the link between behaviour and rapid evolutionary dynamics in a field cricket. *J. Anim. Ecol.* **87**, 623-633 (2018).
13. Rayner, J. G., Aldridge, S., Montealegre, Z. F. & Bailey, N. W. A silent orchestra: convergent song loss in Hawaiian crickets is repeated, morphologically varied, and widespread. *Ecology* **100**, e02694 (2019).
14. Gray, D. A., Kunerth, H. D., Zuk, M., Cade, W. H. & Balenger, S. L. Molecular biogeography and host relations of a parasitoid fly. *Ecol. Evol.* **9**, 11476-11493 (2019).
15. Bailey, N. W. & Zuk, M. Changes in immune effort of male field crickets infested with mobile parasitoid larvae. *J. Insect Physiol.* **54**, 96-104 (2008).
