# Supplementary material for: Temporal genomics in Hawaiian crickets reveals compensatory intragenomic coadaptation during adaptive evolution
Source: Nat Commun. 2024 Jun 12;15:5001. doi: 10.1038/s41467-024-49344-4 (PMC11169259; doi:10.1038/s41467-024-49344-4)
Supplement: Supplementary file 3 — Reporting Summary [file 41467_2024_49344_MOESM3_ESM.pdf]

nature portfolio

Corresponding author(s): Xiao Zhang, Nathan W. Bailey

Last updated by author(s): May 9, 2024

Reporting Summary

Nature Portfolio wishes to improve the reproducibility of the work that we publish. This form provides structure for consistency and transparency in reporting. For further information on Nature Portfolio policies, see our Editorial Policies and the Editorial Policy Checklist.

Statistics

For all statistical analyses, confirm that the following items are present in the figure legend, table legend, main text, or Methods section.

n/a	Confirmed	
☐	☒	The exact sample size (*n*) for each experimental group/condition, given as a discrete number and unit of measurement
☐	☒	A statement on whether measurements were taken from distinct samples or whether the same sample was measured repeatedly
☐	☒	The statistical test(s) used AND whether they are one- or two-sided<br>*Only common tests should be described solely by name; describe more complex techniques in the Methods section.*
☒	☐	A description of all covariates tested
☐	☒	A description of any assumptions or corrections, such as tests of normality and adjustment for multiple comparisons
☐	☒	A full description of the statistical parameters including central tendency (e.g. means) or other basic estimates (e.g. regression coefficient) AND variation (e.g. standard deviation) or associated estimates of uncertainty (e.g. confidence intervals)
☐	☒	For null hypothesis testing, the test statistic (e.g. *F*, *t*, *r*) with confidence intervals, effect sizes, degrees of freedom and *P* value noted<br>*Give P values as exact values whenever suitable.*
☒	☐	For Bayesian analysis, information on the choice of priors and Markov chain Monte Carlo settings
☐	☒	For hierarchical and complex designs, identification of the appropriate level for tests and full reporting of outcomes
☒	☐	Estimates of effect sizes (e.g. Cohen's *d*, Pearson's *r*), indicating how they were calculated

Our web collection on statistics for biologists contains articles on many of the points above.

Software and code

Policy information about availability of computer code

Data collection	Oxford Nanopore Technologies (ONT) libraries were sequenced on the ONT PromethION platform. The ONT basecalling was conducted according to the manufacturer's protocols. Whole genome re-sequencing data were generated using the Illumina HiSeq X platform at Edinburgh Genomics. Demultiplexing and adaptor trimming were carried out using bcl2fastq (v2.20, Illumina), with an allowance of 1 mismatch during the assignment of reads to barcodes.
Data analysis	We used wtdbg2 (v.2.5), minimap2 (2.17-r941), wtpoa-cns (19830203), BWA-MEM(v0.7.17), 3D-DNA (v180419), BWA (v0.7.17), SAMtools (v1.9), Picard Tools (v2.26.2), ALL-HiC (0.9.13), Purge Haplotigs (v1.1.2), TGS-GapCloser (v 1.0.3), Pilon (v1.23), minimap2 (2.17-r941), RepeatModeler (v2.0.1), RECON (v1.08), RepeatScout (v1.0.6), RepeatProteinMask (v4.1.0), TEclass (v2.1.3), TRF (v4.09), SNAP (2013_11_29), Glimmer-HMM (3.0.4), GENEID (v 1.4), BRAKER (v 2.1.5), HISAT2 (v 2.2.0), SAMTOOLS (v1.9), GeneMark-ET (v4, January 2020), AUGUSTUS (v3.3.3), StringTie (v 2.1.2), edgeR (v3.30.3), BLAST (v2.9.0), BLAST2GENE (version 17), GENEWISE2 (v2-4-1), Liftoff (v1.6.3), EVidenceModeler (EVM), Program to Assemble Spliced Alignments (PASA, v 2.4.1), assembly-stats (https://github.com/sanger-pathogens/assembly-stats), Picard (v2.23.6), GATK (v3.8), PLINK (v1.90b6.12), VCFtools (v0.1.16), Beagle (v5.4), Selscan (v2.0.2), norm (v1.2.1), LDheatmap (v1.0-6), PHYLIP (v 3.697), MEGA (v10.0.5), EIGENSOFT (v7.2.1), ADMIXTURE (v1.3.0), prepDE.py3 (http://ccb.jhu.edu/software/stringtie/dl/prepDE.py3), R (v 4.0.3), edgeR (v 3.32.0), crbBlast (0.6.6, blast v2.5.0), Venn diagram tool (https://bioinformatics.psb.ugent.be/webtools/Venn/) in this study (see Methods).

For manuscripts utilizing custom algorithms or software that are central to the research but not yet described in published literature, software must be made available to editors and reviewers. We strongly encourage code deposition in a community repository (e.g. GitHub). See the Nature Portfolio guidelines for submitting code & software for further information.

Data

Policy information about availability of data

All manuscripts must include a data availability statement. This statement should provide the following information, where applicable:

- Accession codes, unique identifiers, or web links for publicly available datasets
- A description of any restrictions on data availability
- For clinical datasets or third party data, please ensure that the statement adheres to our policy

The genome sequencing reads, Hi-C reads, and the genome assembly for T. oceanicus have been deposited in the European Nucleotide Archive with the accession numbers PRJEB63577 [https://www.ebi.ac.uk/ena/browser/view/PRJEB63577] and CAXIVR010000000 [https://www.ebi.ac.uk/ena/browser/view/CAXIVR010000000]. The whole-genome re-sequencing reads that support the findings of this study have been deposited in the European Nucleotide Archive with the accession numbers PRJEB63577 [https://www.ebi.ac.uk/ena/browser/view/PRJEB63577] and PRJEB39125 [https://www.ebi.ac.uk/ena/browser/view/PRJEB39125]. Previously published RNA-seq data have been deposited in the European Nucleotide Archive under accession numbers PRJEB27235 [https://www.ebi.ac.uk/ena/browser/view/PRJEB27235], PRJNA636298 [https://www.ebi.ac.uk/ena/browser/view/PRJNA636298], PRJNA344019 [https://www.ebi.ac.uk/ena/browser/view/PRJNA344019], PRJNA283744 [https://www.ebi.ac.uk/ena/browser/view/PRJNA283744], and PRJEB40088 [https://www.ebi.ac.uk/ena/browser/view/PRJEB40088]. Following public databases were used in this study: Dfam consensus database (v.3.1) [https://www.dfam.org/releases/Dfam_3.1/], NR database [https://www.ncbi.nlm.nih.gov/refseq/about/nonredundantproteins/], GO database [http://geneontology.org/], KEGG database [https://www.kegg.jp], Swiss-Prot and TrEMBL databases [http://www.uniprot.org/downloads]. Source data are provided with this paper.

Research involving human participants, their data, or biological material

Policy information about studies with human participants or human data. See also policy information about sex, gender (identity/presentation), and sexual orientation and race, ethnicity and racism.

Reporting on sex and gender	n/a
Reporting on race, ethnicity, or other socially relevant groupings	n/a
Population characteristics	n/a
Recruitment	n/a
Ethics oversight	n/a

Note that full information on the approval of the study protocol must also be provided in the manuscript.

Field-specific reporting

Please select the one below that is the best fit for your research. If you are not sure, read the appropriate sections before making your selection.

☐ Life sciences ☐ Behavioural & social sciences ☒ Ecological, evolutionary & environmental sciences

For a reference copy of the document with all sections, see nature.com/documents/nr-reporting-summary-flat.pdf

Ecological, evolutionary & environmental sciences study design

All studies must disclose on these points even when the disclosure is negative.

Study description	We produced a new chromosome-level de novo genome assembly for T. oceanicus using Oxford Nanopore PromethION long reads from a female homozygous for the normal-wing genotype, from laboratory stock originally derived from the Kauai population. We polished this assembly with Illumina short reads and anchored it with Illumina paired-end reads from a high-throughput chromosome conformation capture (Hi-C) library. We annotated this genome assembly by identifying repetitive sequences and predicted protein-coding genes. Then we performed whole genome re-sequencing, conducted genome-wide association analyses, and tested for linkage disequilibrium around a putative locus underlying an adaptive, 'flatwing', male phenotype. We also performed gene expression analyses related to two traits in T. oceanicus: plasticity to singing vs. silent acoustic environments, and immunological responses to parasitoid infestation. We tested for compensatory genomic evolution provoked by the rapid spread of flatwing by developing a combinatorial enrichment approach and using bottom-up and top-down methods with gene expression data.
Research sample	For the de novo genome assembly based on PromethION long reads, we used one female field cricket (Teleogryllus oceanicus) from a laboratory line derived from Kauai and homozygous for the normal-wing genotype, which had experienced multiple generations of laboratory inbreeding. We improved single-base accuracy by polishing with previously-published Illumina HiSeqX short reads from a male of the same stock population. We used third individual from the same stock population to construct Hi-C libraries. For analyses based on whole genome resequencing data, 47 male T. oceanicus field crickets were used. Among these crickets, thirty flatwing males were collected from the same wild Kauai population in 2012, 2017, and 2018 (10 each year), in time intervals spanning the transition from a social environment with song to a silent social environment. Ten normal-wing male Kauai field crickets from laboratory stocks derived from the same field location were also used. The latter individuals were taken from existing laboratory stocks because of the extreme scarcity of normal-wing males in the Kauai population (or indeed absence in later years). In addition,

	seven Australian field crickets were resequenced as comparators, because Australian populations are not under selection pressure from the parasitoid fly. Data from 114 RNA-seq samples were obtained from five previous studies (Supplementary Data 2), which had been deposited in the European Nucleotide Archive (ENA) under accession numbers PRJEB27235, PRJNA636298, PRJNA344019, PRJNA283744, and PRJEB40088.
Sampling strategy	Three field crickets (Teleogryllus oceanicus) from a laboratory line derived from Kauai and homozygous for the normal-wing genotype, which had experienced multiple generations of laboratory inbreeding were used to generate the reference genome assembly (see Methods). Thirty flatwing males were collected from the same wild Kauai population in 2012, 2017, and 2018 (10 each year). Whole-genome re-sequencing data from 7 normal-wing Australian individuals and 10 normal-wing lab-reared Kauai individuals were used for comparison. Data from 114 RNA-seq libraries were used for gene predictions, which covered four tissues, three populations, both sexes, and two wing morph genotypes (Supplementary Data 2). Data from 66 RNA-seq samples covering two tissues (neural and general body tissue), two populations, both sexes, and both wing morph genotypes were used as an alternative to screening panels of complex physiological characters and validate the functions of candidate genes (Fig. 5d, Supplementary Data 2). Sample sizes for resequencing and RNAseq reflected trade-offs between sequencing cost, read depth and statistical power.
Data collection	To avoid biases arising from local relatedness structure, samples for whole genome re-sequencing were collected haphazardly within each population by Nathan W. Bailey and Jack G. Rayner with assistance from Katherine Holmes, Tony Ly, Peter Moran, Sonia Pascoal, Michael Ritchie, John Rotenberry, Will Schneider, Suzanne Vardy, and Marlene Zuk. For the chromosome-level de novo genome assembly, Nathan W. Bailey selected lab-reared samples randomly from existing laboratory stock derived from Kauai. Previously published RNA-seq data were downloaded from the European Nucleotide Archive using accession numbers PRJEB27235, PRJNA636298, PRJNA344019, PRJNA283744, and PRJEB40088, which were identified in the main text and Supplementary Data 2.
Timing and spatial scale	Timings of sampling for the study are described in Supplementary Data 1. Among 47 crickets used for population genetic analyses, thirty flatwing males were collected in Kauai. Ten normal-wing male Kauai field crickets from laboratory stocks derived from the same field location and seven Australian field crickets were used for comparison.
Data exclusions	No data were excluded from the analyses.
Reproducibility	We performed multiple analyses for each set of conclusions, e.g. evaluating genome assembly using four independent techniques (BUSCO, whole genome re-sequencing data alignment, RNA-seq data mapping, comparison with other published field cricket genome assemblies), e.g. test for compensatory genomic evolution provoked by the rapid spread of flatwing using selection-based and differentiation-based methods, and e.g. test for functionally association between genomic signatures of selection and the transition to total silence using bottom-up and top-down methods with gene expression data. The combination of multiple lines of evidence supports our conclusion that the findings are reproducible.
Randomization	We did not apply experimental treatments in this study, so randomization was not implemented in this context. We made efforts to not bias sampling by relatedness structure within populations, and when laboratory samples were contrasted with in-situ samples from populations in nature, this was clearly identified in the main text.
Blinding	All samples were numbered and subject to sequencing by staff members blind to our sampling strategy. Blinding was also used during analysis.

Did the study involve field work? ☐ Yes ☒ No

Reporting for specific materials, systems and methods

We require information from authors about some types of materials, experimental systems and methods used in many studies. Here, indicate whether each material, system or method listed is relevant to your study. If you are not sure if a list item applies to your research, read the appropriate section before selecting a response.

Materials & experimental systems

n/a	Involved in the study	
☒	☐	Antibodies
☒	☐	Eukaryotic cell lines
☒	☐	Palaeontology and archaeology
☐	☒	Animals and other organisms
☒	☐	Clinical data
☒	☐	Dual use research of concern
☒	☐	Plants

Methods

n/a	Involved in the study	
☒	☐	ChIP-seq
☒	☐	Flow cytometry
☒	☐	MRI-based neuroimaging

Animals and other research organisms

Policy information about studies involving animals; ARRIVE guidelines recommended for reporting animal research, and Sex and Gender in Research

Laboratory animals	Genome assembling, RNAseq and GWAS involving normal-wing Kauai males (L) used lab stocks. Supplementary Data 3 provides further sampling details.

Wild animals	Single hind legs of male samples (T. oceanicus) were collected from Australia and Hawaii and preserved in 100% ethanol (Supplementary Data 1 and 3) for gDNA extraction.
Reporting on sex	Whole genome resequencing used male individuals due to the need to identify their wing phenotype based on externally visible characteristics (the adaptive phenotype under investigation is male-limited). Sex was identified by checking for the presence or absence of an ovipositor. In previously-published RNAseq data examined in this study, both sexes were included in the experimental design evaluating responses to acoustic signals as both sexes perceive sound. Only males were used in RNAseq experiments examining responses to parasitoid infestation: the parasitoids locate hosts by eavesdropping on host song, male crickets sing but females do not.
Field-collected samples	Tissue samples from thirty flatwing males were collected from the same wild Kauai population in 2012, 2017, and 2018 (10 each year), in time intervals spanning the transition from a social environment with song to a silent social environment. Ten normal-wing male Kauai field crickets from laboratory stocks derived from the same field location were also used. The latter individuals were taken from existing laboratory stocks because of the extreme scarcity of normal-wing males in the Kauai population (or indeed absence in later years). In addition, seven Australian field crickets were resequenced for comparison, because the Australian populations are not under selective pressure from the parasitoid fly. Among all 47 samples, resequencing data from 27 individuals was previously published. The 20 new individuals in this study were resequenced using identical procedures. The study species is not subject to ethical regulations and approval of study protocols was not required.<br><br>The research complied with all relevant ethical regulations. Export permission was obtained from Australian Department of Sustainability, Environment, Water, Population and Communities (WT2012-6184). Although permits were not required for non-native invertebrate sampling at Hawaiian field sites, we obtained collecting permission from landowners and the Kauai Agricultural Research Centre. Access was gained by driving and walking.
Ethics oversight	Approval of study protocols was not required for this species. The research complied with all relevant ethical regulations. Export permission was obtained from Australian Department of Sustainability, Environment, Water, Population and Communities (WT2012-6184). Although permits were not required for non-native invertebrate sampling at Hawaiian field sites, we obtained collecting permission from landowners and the Kauai Agricultural Research Centre. Access was gained by driving and walking.

Note that full information on the approval of the study protocol must also be provided in the manuscript.
